# Reasons for low utilisation of public facilities among households with hypertension: analysis of a population-based survey in India

**DOI:** 10.1136/bmjgh-2018-001002

**Published:** 2018-12-20

**Authors:** Stephanie A Kujawski, Hannah H Leslie, Dorairaj Prabhakaran, Kavita Singh, Margaret E Kruk

**Affiliations:** 1 Department of Epidemiology, Columbia University Mailman School of Public Health, New York, New York, USA; 2 Department of Global Health and Population, Harvard T.H. Chan School of Public Health, Boston, Massachusetts, USA; 3 Centre for Chronic Disease Control, New Delhi, India; 4 Public Health Foundation of India, Gurgaon, India; 5 London School of Hygiene and Tropical Medicine, London, UK

**Keywords:** cardiovascular disease, hypertension, diabetes, quality of care, India

## Abstract

**Introduction:**

In India, for most patients, primary healthcare remains the intended entry point for the management of non-communicable disease risk factors. The extent and determinants of non-utilisation of public primary care among households with hypertension are not well examined. We explored health facility utilisation patterns and reasons for non-utilisation of public facilities in 21 states and union territories in India, with a focus on hypertension.

**Methods:**

We used data from the 2012–2013 District Level Household and Facility Survey. We examined the self-reported usual source of care for all households, households with hypertension and─to understand multimorbidity for those with hypertension─households with hypertension and diabetes. Hypertension was defined by self-reported diagnosis or measurement of systolic blood pressure ≥140 mm Hg or diastolic blood pressure ≥90 mm Hg. Diabetes was defined by self-reported diagnosis or fasting blood glucose level ≥ 7.0 mmol/L or non-fasting blood glucose level ≥ 11.1 mmol/L. We assessed facility utilisation choice and reasons for non-utilisation of public facilities by household with the presence of hypertension alone and hypertension with diabetes.

**Results:**

In 336 305 households, 37.6% (N=126 597) had at least one household member with hypertension, while 15.9% (N=53 385) had members with hypertension and diabetes. 20.0% of households sought care at public primary clinics, 29.9% at public hospitals and 48.3% at private facilities. Choice of private facilities increased with the burden of disease. Households with hypertension only and hypertension and diabetes cited quality reasons for non-utilisation of public facilities more than households without hypertension.

**Conclusion:**

Households, particularly those with hypertension, chose private over public primary facilities for usual care. Quality of care was an important determinant of facility choice in households with hypertension and diabetes. With the increase in hypertension and cardiovascular disease in India, quality of public primary healthcare must be addressed for current policy to become reality.

**Key questions**

**What is already known?**

- The non-communicable disease burden in India is increasing, with cardiovascular disease now the number one cause of death.
- For most patients, primary care facilities in India are the intended entry point for the prevention and diagnosis of non-communicable disease risk factors, such as hypertension, yet health system users in low-income and middle-income countries are known to use private facilities in search of better quality of care.

**What are the new findings?**

- In this population-based survey of 336 305 households in 21 state and union territories in India, households chose private facilities for care, with this choice increasing with the burden of hypertension and hypertension–diabetes multimorbidity.
- Households with hypertension only and with both hypertension and diabetes were more likely to cite quality of care as a reason for non-utilisation public facilities than households without hypertension.

**What do the new findings imply?**

- Quality of health care is an important determinant of facility choice in households with hypertension and choice of private facilities signals that the primary public health system is failing to respond to the changing health needs of the population.

## Introduction

India is undergoing an epidemiological transition, with the burden of non-communicable diseases increasing significantly over the past 25 years.^1^ Cardiovascular disease is now the number one cause of death, accounting for 28.1% of deaths in India and 14.1% of life-years lost.^2^ Hypertension is one of the primary risk factors for cardiovascular disease, with 54.6% of cardiovascular disease disability-adjusted life-years attributable to high systolic blood pressure.^2^ Those with hypertension and additional cardiovascular disease risk factors, such as diabetes, are at especially elevated risk for cardiovascular disease.^3^ Hypertension is on the rise in India, with an estimated hypertension prevalence of 26.5% in adults 18 years and older.^4^

For most patients, primary healthcare in India remains the intended entry point for the prevention and diagnosis of hypertension. The National Programme for the Prevention and Control of Diabetes, Cardiovascular Diseases and Stroke, initiated in 2010, promotes public health system strengthening at all levels for opportunistic detection and screening for non-communicable diseases.^5^ In 2016, the National Health Mission and the Ministry of Health and Family Welfare in India released operational guidelines for the public health system for non-communicable diseases that recommend routine screening for the entire population over 30 years of age, including blood pressure assessment.^6^ The health and wellness centres proposed as part of the recently launched Ayushman Bharat programme will serve as the first entry point for the management of non-communicable disease risk factors and will be responsible for screening and routine management of hypertension as part of comprehensive primary care.^6 7^

Primary care can be an effective and cost-effective platform for tackling non-communicable diseases.^8 9^ However, public primary facilities in India often lack the human resources, diagnostic equipment and drugs to diagnose and treat the nearly 200 million adults living with hypertension in India.^10–13^ Patients with these conditions also report long wait times and insufficient time with providers at the public primary care level in qualitative studies.^12 13^ As such, patients may choose to seek care at private or higher level health facilities. A population representative study of older individuals in India found that those with diabetes or hypertension were even more likely to use private care than other adults.^14^ However, no prior studies quantitatively address reasons for choice of facilities among adults with hypertension at a national scale.

Clinic choice and non-utilisation of public primary healthcare is an important indication of the (low) value that people place on nearby or first line health services.^15^ However, non-utilisation of public primary health facilities incurs individual as well as health system costs. Indian adults spent a median of US$11.80 out of pocket when accessing private health clinics compared with US$1.00 for public or other health centres.^14^ High healthcare costs driven by such expenditures are a leading cause of poverty in India.^16^ While bypassing poor quality facilities may lead people to better quality care, this is not always the case since technical care quality is not fully observable to non-experts.^15 17^ Further, relying on referral facilities for routine care can undermine continuity of care: utilisation of higher-level facilities for routine care is often for one-off visits without continued patient follow-up or management of care. From the perspective of health system financing and design, investing in primary care services that are not used is inefficient, while overuse of secondary facilities may blunt their capacity to provide adequate and timely care for complex or acute conditions. Prevalence and reasons for bypassing or non-utilisation of public primary care facilities are likely to differ based on the health need, as patients balance cost and expected benefit specific to a given condition.^18^ Policymakers need evidence on reasons for non-utilisation public primary care facilities to inform India’s response to the growing epidemic of hypertension. To date, to our knowledge, no studies have considered reasons for health facility choice among people with hypertension at the population level in India.

To understand health system preferences as India prepares to tackle the growing burden of non-communicable diseases, this paper explores health facility utilisation patterns and reasons for non-utilisation of public facilities among households in 21 states and union territories in India, with a particular focus comparing households with hypertension to households without hypertension. To understand the influence of co-morbidity, a secondary analysis examined these aims among households with both hypertension and diabetes.

## Methods

### Study design and sample

We used publicly available data from the fourth cycle of the District Level Household and Facility Survey (DLHS-4) implemented by the International Institute of Population Sciences in India. The DLHS is a population-based survey conducted in states and union territories not included in India’s Annual Health Survey. The DLHS-4 was conducted from 2012 to 2013 in 21 states and union territories in India using a multi-stage stratified sampling design.^19^ Generally, within each state, each district was stratified into urban and rural areas. Then, a two-stage sampling frame was used with the primary sampling unit as urban blocks or rural census villages and the secondary sampling unit as the household.^20^ The methods are described elsewhere.^19 20^ The household head was interviewed about the households’ characteristics.^21^ In addition, consenting individual household members available at the time of the household interview aged 18 and older had their blood pressure measurements taken and blood collected for blood glucose levels. Individuals were asked if they had consumed any food or liquid prior to the blood draw. This analysis was restricted to households where at least one household member provided complete individual-level biomarker data for measures of hypertension and diabetes.

### Measures

The main measures of interest were household hypertension status and health facility utilisation choice. Households were categorised into three distinct groups: households without hypertension (no members with hypertension), households with hypertension only (at least one member with hypertension, no members with diabetes) and households with both hypertension and diabetes (at least one member with hypertension and at least one member with diabetes). For households with hypertension only, at least one household member with chronic disease had to have self-reported a past hypertension diagnosis or have systolic blood pressure of ≥140 mm Hg or diastolic blood pressure of ≥90 mm Hg based on the mean of two blood pressure measurements and have no household members with diabetes.^22^ For households with both hypertension and diabetes, at least one household member had to meet the hypertension criteria above and at least one household member (same or different) had to report past diagnosis of diabetes or have a blood glucose level of ≥ 7.0 mmol/L for those who reported not consuming food or liquid prior to the test (fasting blood glucose) or a blood glucose level of ≥ 11.1 mmol/L for those who reported consuming any food or liquid consumption prior to the test (non-fasting blood glucose).^4^

Household choice of public or private health facilities was assessed with a question asking where household members generally seek care when they are sick. Facilities were categorised as public primary healthcare facility (subcentre, primary health centre, community health centre, urban health centre, dispensary/clinic), public hospital, private facility and other (non-medical shop, home treatment, other).

Households that chose private facilities were asked why they did not choose a public facility using a multiresponse question. We categorised these reasons for non-utilisation of public facilities into three domains^23 24^: access (no public facility available, facility too far away, not aware of any facility), technical quality (poor quality of care, doctor not available, drugs not available, health personnel often absent, no adequate infrastructure) and non-technical quality (wait time too long, facility timing not convenient, distrust).

To describe the characteristics of the household, we included a mix of demographic variables based on the relevant literature^25 26^: mean age of the household, age of the head of household, household has a member less than 5 years of age, household has a member ≥65 years of age, mean number of household members, mean number of male members, mean number of female members, sex of the head of household, religion (Hindu vs other religions), household has health insurance, household has a below the poverty line card, socioeconomic status and household location (rural vs urban). Socioeconomic status was measured using a principal component analysis of 14 household asset variables (main source of lighting, housing structure (three variables), fuel used for cooking and ownership of a radio, television, telephone, sewing machine, bicycle, motorcycle, car, tractor).^27^ The results were split into quintiles, with the lowest 40% categorised as poor.^27^

### Statistical analysis

The survey included weights to account for sampling within each state. To generate representative estimates across states, we scaled the weights based on the 2011 state population.^28–30^ Demographic characteristics of all households, households without hypertension, households with hypertension only and households with hypertension and diabetes were summarised with descriptive statistics. Next, using descriptive statistics, facility utilisation choice (public primary facility, public hospital, private facilities, other) was assessed by household non-communicable disease status. To account for possible differences in availability of health facilities by location, these results were then stratified by household location (rural vs urban). A secondary analysis stratified facility utilisation choice among households with hypertension only by state/union territory. As households that chose private facilities could provide more than one reason for non-utilisation of public facilities, the total number of reasons provided was summed. We then calculated the relative importance of each reason for non-utilisation of public facilities and by domain (access, technical quality, non-technical quality). We compared reasons for non-utilisation of public health facilities between households without hypertension and (1) households with hypertension only and (2) households with hypertension and diabetes among all households, among poor households and among households with no health insurance by calculating an absolute difference and using chi-square tests. These analyses were also stratified by household location. Analyses were conducted in Stata V.14.

## Results

A total of 378 487 households were included in the DLHS-4, of which 378 280 had weight information available. Of the 378 280 households, 378 195 had a household member ≥18 years of age and were therefore eligible for biomarker screening. Eighty-nine per cent of the eligible households (N=336 305) had complete hypertension and diabetes biomarker data available.

The majority of households were of Hindu religion (N=263 576, 78.4%) and had a male head of household (N=287 224, 85.4%) (table 1). About a quarter of households had health insurance (N=94193, 28.0%) and 42.2% were categorised as poor (N=141 239). About 38% (37.6%, N=126 597) of households had at least one household member with hypertension only, while 15.9% (N=53 385) of households had both hypertension and diabetes. A larger proportion of households with hypertension and diabetes had health insurance (N=15 508, 29.1%) and a smaller proportion were categorised as poor (N=14 801, 27.8%) compared with households without hypertension (insured: N=43 891, 28.1%; poor: N=75 423, 48.5%). Households with non-communicable disease risk factors were generally older, with the mean age of the household increasing with the burden of non-communicable diseases. About a third (30.3%) of households with hypertension only and 40.7% of households with hypertension and diabetes had a household member ≥65 years of age compared with 17.3% of households without hypertension. Data for at least one descriptive variable were missing for 0.08% of the analytic sample.

**Table 1:** Demographic characteristics of households with biomarker data in the District Level Household and Facility Survey-4 by chronic disease status, 2012–2013

Household demographics	All households (N=336 305)		Households without hypertension (N=156 323)		Households with hypertension only (N=126 597)		Households with hypertension and diabetes (N=53 385)	
	N	%*	N	%*	N	%*	N	%*
Mean age of household members (mean, SD)	33.5	12.9	30.7	12.3	35.0	12.9	38.0	12.6
Mean age of head of household (mean, SD)	49.2	13.9	45.9	13.2	50.9	13.8	54.8	13.4
Any household member <5 years of age	95 352	28.4	47 672	30.5	33 393	26.4	14 288	26.8
Any household member ≥65 years of age	87 183	25.9	27 052	17.3	38 410	30.3	21 721	40.7
Mean household size (mean, SD)	4.5	2.2	4.2	1.9	4.5	2.2	4.9	2.6
Mean number of males in household (mean, SD)	2.2	1.3	2.1	1.2	2.3	1.3	2.4	1.5
Mean number of females in household (mean, SD)	2.2	1.3	2.1	1.2	2.3	1.3	2.5	1.5
Sex of head of household is male	287 224	85.4	132 993	85.1	107 895	85.2	46 336	86.8
Hindu religion (vs all other religions)	263 576	78.4	124 294	79.5	97 970	77.4	41 313	77.4
Household has health insurance	94 193	28.0	43 891	28.1	34 795	27.5	15 508	29.1
Household has a below the poverty line card	144 136	42.8	69 569	44.5	54 008	42.7	20 560	38.5
Household has electricity	320 438	95.3	147 285	94.2	121 167	95.7	51 987	97.4
Household has a bicycle	152 722	45.4	71 675	45.9	56 373	44.5	24 675	46.2
Poor (lowest 40%)	141 594	42.1	75 657	48.4	51 168	40.4	14 769	27.7
Rural household location	208 099	61.9	101 408	64.9	78 927	62.3	27 764	52.0

*Sampling weight proportion/SD.

Table 2 presents self-reported facility utilisation for usual care by the non-communicable disease status of the household. Overall, households did not utilise the public primary health system, with only 20.0% of all households generally seeking care when sick at public primary health facilities. The majority of households chose private facilities (N=162 382, 48.3%). In comparison to households without hypertension (45.6%), there was increasing choice of the private sector with the burden of non-communicable disease risk factors: 49.2% of households with hypertension only and 54.1% of households with the double burden of hypertension and diabetes chose private sector facilities. When stratified by household location, there was a similar pattern of choice for private facilities overall and among households with non-communicable disease risk factors. However, a higher percentage of urban households chose private sector (55.2%) facilities than rural households (44.1%). Among households with hypertension only, the proportion of people choosing public primary care varied across states, from a low of 3.8% in Haryana to a high of 57.2% in Meghalaya (online supplementary figure 1); Meghalaya was the only state with over half of the population seeking care in public primary facilities.

10.1136/bmjgh-2018-001002.supp1 Supplementary data

**Table 2:** Facility utilisation patterns of households with biomarker data in the District Level Household and Facility Survey-4 by chronic disease status, 2012–2013

	All households (N=336 305)		Households without hypertension (N=156 323)		Households with hypertension only (N=126 597)		Households with hypertension and diabetes (N=53 385)	
	N	%*	N	%*	N	%*	N	%*
Public primary healthcare facility	67 021	20.0	34 104	21.8	29 904	19.7	8013	15.0
Public hospital	100 379	29.9	47 378	30.3	37 194	29.4	15 807	29.6
Private facility	162 382	48.3	71 226	45.6	62 289	49.2	28 867	54.1
Other (non-medical shop, home treatment, other)	6233	1.9	3489	2.2	2111	1.7	633	1.2
Missing	289	0.1	126	0.1	98	0.1	65	0.1
Rural household location	(N=208 099)		(N=101 408)		(N=78 926)		(N=27 764)	
Public primary healthcare facility	51 400	24.7	26 745	26.4	19 089	24.2	5566	20.1
Public hospital	59 666	28.7	29 135	28.8	22 369	28.3	8162	29.4
Private facility	91 693	44.1	42 442	41.9	35 710	45.3	13 541	48.8
Other (non-medical shop, home treatment, other)	5185	2.5	2997	3.0	1708	2.2	480	1.7
Missing	155	0.1	89	0.1	51	0.1	15	0.1
Urban household location	(N=128 206)		(N=54 915)		(N=47 671)		(N=25 621)	
Public primary healthcare facility	15 621	12.2	7358	13.4	5816	12.2	2447	9.6
Public hospital	40 713	31.8	18 243	33.2	14 825	31.1	7645	29.9
Private facility	70 690	55.2	28 784	52.5	26 579	55.8	15 326	59.9
Other (non-medical shop, home treatment, other)	1048	0.8	492	0.9	403	0.9	153	0.8
Missing	134	0.1	38	0.1	47	0.1	49	0.2

*Sampling weight proportion/SD.

**Figure 1:**
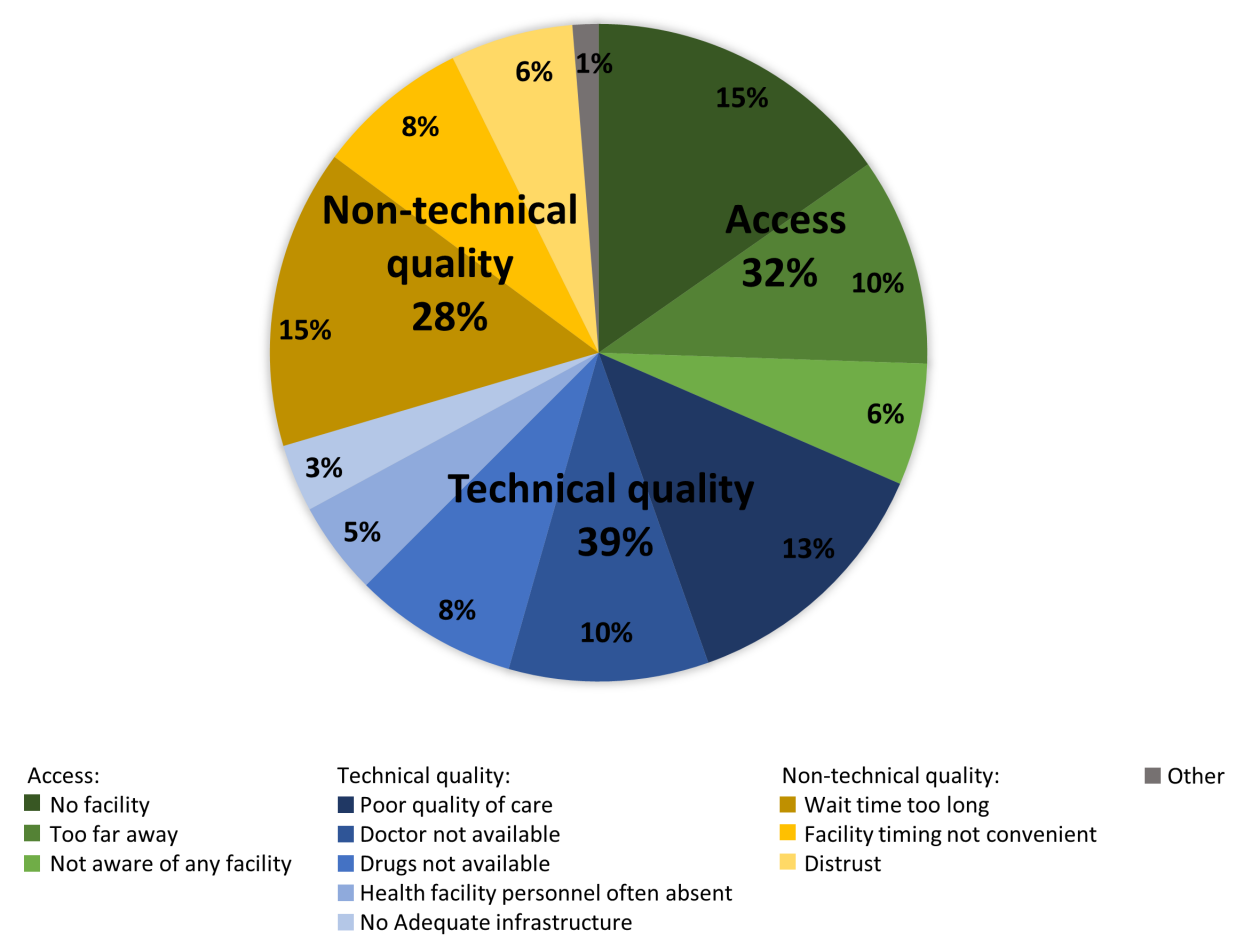
Among households that chose private facilities, reasons for non-utilisation of public facilities (N=162 382 households, N=447 881 reasons).

Figure 1 shows the distribution of reasons for non-utilisation of public facilities. Technical quality accounted for the largest proportion of the reasons for non-utilisation (39%), followed by access (32%) and non-technical quality (28%). The top individual reasons for non-utilisation of public facilities were no public facility available (15%), too long of a wait time (15%), poor quality of care (13%), facility too far away (10%) and no doctor available (10%).

Small but statistically significant differences (at p<0.01) in reasons for non-utilisation of public facilities were apparent based on household health status (table 3). Compared with households without hypertension, households with hypertension only were more likely to cite quality (technical or non-technical) as a reason for non-utilisation of public facilities (79.5% vs 78.5%). The difference was even larger for households with both hypertension and diabetes (80.8% citing a quality-related reason vs 78.5% of households without hypertension). Households with both hypertension and diabetes were less likely to mention any access reason (65.5%) compared with households without hypertension (68.1%).

**Table 3:** Among households that chose private facilities, reasons for non-utilisation of public facilities, District Level Household and Facility Survey-4, 2012–2013 (N=162 382)

	Households without hypertension		Households with hypertension only		Difference in %	χ^2^ p-value	Households with hypertension and diabetes		Difference in %	χ^2^ p value
	N	%*	N	%*			N	%*		
All households	N=71 226		N**=**62 289				N**=**28 867			
Any access reason	48 521	68.1	42 769	68.7	0.60	0.12	18 916	65.5	−2.60	<0.0001
Any technical quality reason	42 510	59.7	38 461	61.8	2.10	<0.0001	17 632	61.1	1.40	0.004
Any non-technical quality reason	40 667	57.1	36 307	58.3	1.20	0.001	17 437	60.4	3.30	<0.0001
Any quality reason (technical or non-technical quality)	55 912	78.5	49 524	79.5	1.00	0.001	23 333	80.8	2.30	<0.0001
Average number of reasons provided (range: 0–12)	2.7		2.9				2.9			
Poor households	N=27 619		N=19 709				N=5553			
Any access reason	19 615	71.0	14 157	71.8	0.80	0.17	3904	70.3	−0.70	0.46
Any technical quality reason	16 612	60.2	12 131	61.6	1.40	0.04	3343	60.2	0.00	0.96
Any non-technical quality reason	14 977	54.2	10 728	54.4	0.20	0.76	3096	55.8	1.60	0.14
Any quality reason (technical or non-technical quality)	21 349	77.3	15 262	77.4	0.10	0.81	4354	78.4	1.10	0.20
Average number of reasons provided (range: 0–12)	2.7		2.8				2.8			
Households with no health insurance	N=51 313		N=45 464				N=20 681			
Any access reason	35 274	68.7	31 731	69.8	1.10	0.007	13 606	65.8	−2.90	<0.0001
Any technical quality reason	30 221	58.9	27 603	60.7	1.80	<0.0001	12 452	60.2	1.30	0.02
Any non-technical quality reason	29 159	56.8	26 304	57.9	1.10	0.01	12 416	60.0	3.20	<0.0001
Any quality reason (technical or non-technical quality)	39 880	77.7	35 713	78.6	0.90	0.02	16 578	80.2	2.50	<0.0001
Average number of reasons provided (range: 0–12)	2.7		2.9				2.9			

*Sampling weight proportion/SD.

The largest difference in reasons for non-utilisation of public facilities was technical quality for households with hypertension only (cited by 61.8% compared with 59.7% of those without), while the households that also had diabetes, the largest difference was for non-technical quality (60.4% vs 57.1%). These differences were similarly reflected among poor households with hypertension and diabetes and among households with no health insurance. For example, among households with no health insurance, a higher proportion of households with hypertension only mentioned any technical quality reason (60.7% vs 58.9%) and a higher proportion of households with both hypertension and diabetes mentioned any non-technical quality reason (60.0% vs 56.8%) than households without hypertension (table 3). Among rural households, a higher percentage reported any access reason and any technical quality reason than urban households, while urban households reported more non-technical quality reasons (online supplementary table 1).

10.1136/bmjgh-2018-001002.supp2 Supplementary data

## Discussion

In a large population-based household survey in 21 states and union territories in India, the majority of households elected private facilities for usual care; household choice of private facilities increased with the number of risk factors for cardiovascular disease in households with hypertension. Small but consistent differences in reasons for non-utilisation of the public health system suggest that quality of healthcare plays a stronger role in households with cardiovascular disease risk factors. This was particularly the case for uninsured households seeking private sector care, implying a direct financial burden on these households for the high cost of private healthcare. These findings reflect the misalignment between the public health system and people’s needs and expectations, and they provide important insights for health administrators and policymakers to strengthen the public healthcare system.

The choice of private health facilities, particularly among those with hypertension, is consistent with the literature on private sector preference in India.^14^ For example, a study in Karnataka, a state with relatively better healthcare than many of the states in India, found that the primary public health system lacked the medicines and human resources and infrastructure to treat and diagnose non-communicable diseases.^13^ These deficiencies were recognised by health system users, who, similar to participants in this study, cited quality factors such as poor availability and quality of medicines and fragmented healthcare in the public sector, as reasons for using private facilities.^13^

While the majority of participants chose to utilise private facilities, a large proportion sought care from public hospitals. Bypassing the public primary healthcare for secondary or tertiary care is inefficient both to the patient and the health system. Seeking diagnostic services for hypertension and other cardiovascular disease risk factors at hospitals can also inhibit long-term care management, which is crucial to ensuring control of disease progression. Particularly in the context of India’s new health insurance scheme which will provide coverage for hospital care,^31^ the trend towards hospital utilisation for diagnosis of non-communicable disease risk factors may continue. This may overburden the hospital system and detract from the goal of providing screening, diagnosis and treatment services of non-communicable diseases at the primary care level.

We found that among all households, technical quality was the most cited category for non-utilisation of public facilities for general illness, and households with cardiovascular risk factors were more likely to cite quality reasons compared with those without hypertension. Interestingly, households with hypertension and diabetes were less likely to mention access reasons, which may reflect that these households place more emphasis on quality when deciding where to seek care.

Quality healthcare as a motivator for facility choice is similarly reported in other studies in India and elsewhere. In Madhya Pradesh, India, residents travelled outside of their village to seek private healthcare at facilities staffed with qualified providers.^26^ In a study performed in Chhattisgarh, India, the majority of participants bypassed the local primary public healthcare facility when seeking care for a recent illness, and absence of providers and providers’ clinical competence influenced bypassing behaviours.^25^ Quality of care is particularly relevant when it is perceived to significantly impact the health outcome.^32^ Interestingly, in this study, households with hypertension or hypertension and diabetes were more likely to mention any quality reason (technical or non-technical quality) for non-utilisation of public facilities than households without hypertension. These findings may thus reflect the desire to seek health facilities with better quality of care for chronic illnesses. Non-utilisation of the primary care level for quality reasons is well documented in obstetric care,^33–35^ where the risk of maternal or newborn death is higher without the proper quality.

This study has important strengths. To our knowledge, this is the first study to use a large, population-based sample in India to assess reasons for non-utilisation of public facilities among those with hypertension. We used a combination of self-report and biomarker measures to define hypertension and diabetes, which allowed for a comprehensive assessment of cardiovascular disease risk factors at the household level. A few limitations should be noted. First, we limited our sample to households that had both household survey data and complete biomarker data available (online supplementary table 2); moreover, prior diagnosis reported by household members could underestimate prevalence. Second, stated choice of the public or private health sector and reasons for non-utilisation of public facilities were available only at the household level, not the individual level. Therefore, it is possible that individuals with hypertension and diabetes might have provided different reasons for non-utilisation of the public health system than those given at the household level for general illness. Third, we were unable to distinguish private primary facilities from private hospitals in the data, as private sector hospitals in India may represent a facility at the primary care level (online supplementary table 3). Future studies should compare utilisation of public primary and private primary facilities to better understand utilisation patterns at the primary healthcare level. Fourth, all states and territories in India were not included in the DLHS-4, thus, limiting the generalisability of the results to the country level.

10.1136/bmjgh-2018-001002.supp3 Supplementary data

10.1136/bmjgh-2018-001002.supp4 Supplementary data

## Conclusion

Choice of the private health sector and non-utilisation of public primary care are signals that the public primary health system is failing to meet the evolving health needs of the population. National level policies call for the primary public health system to provide screening and management of hypertension. However, this study highlights that quality of care was an important determinant of non-utilisation of public facilities. In the context of these policies and the increase in non-communicable diseases in India, the public primary health system needs to urgently prioritise high quality non-communicable disease care if current policy is to become a reality.
