# Supplementary figures and images for: Reasons for low utilisation of public facilities among households with hypertension: analysis of a population-based survey in India
Source: BMJ Glob Health. 2018 Dec 20;3(6):e001002. doi: 10.1136/bmjgh-2018-001002 (PMC6307571; doi:10.1136/bmjgh-2018-001002)

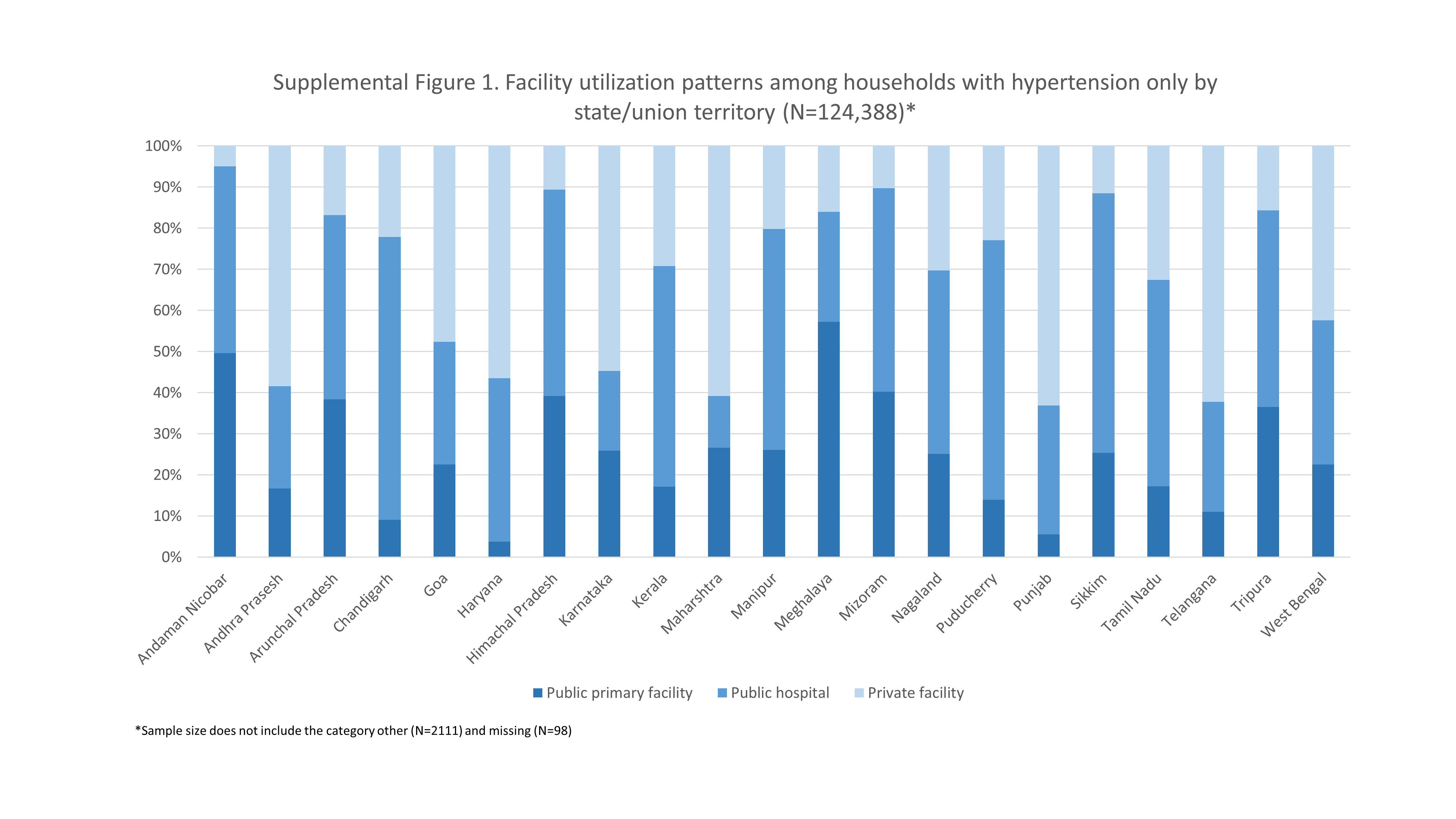

Supplement: Supplementary data [file bmjgh-2018-001002supp001.tif]
