# Supplementary material for: Reasons for low utilisation of public facilities among households with hypertension: analysis of a population-based survey in India
Source: BMJ Glob Health. 2018 Dec 20;3(6):e001002. doi: 10.1136/bmjgh-2018-001002 (PMC6307571; doi:10.1136/bmjgh-2018-001002)
Supplement: Supplementary data [file bmjgh-2018-001002supp002.pdf]

Supplemental Table 1. Among households with preference for private facilities, reasons for non-utilization of public facilities stratified by household location (N=162,382)							
**RURAL HOUSEHOLDS**							
	**Households without hypertension**	**Households with hypertension only**	**Difference in %**	**chi-sq p-value**	**Households with hypertension and diabetes**	**Difference in %**	**chi-sq p-value**
**All households**							
	%[a]	%[a]			%[a]		
Any access reason	70.2	70.7	0.50	0.29	68.2	-2.00	0.002
Any technical quality reason	61.5	63.9	2.40	<0.0001	64.3	2.80	0.0002
Any non-technical quality reason	55.6	56.6	1.00	0.05	58.9	3.30	<0.0001
Any quality reason (technical or non-technical quality)	78.6	79.6	1.00	0.03	81.3	2.70	<0.0001
**Poor households**							
Any access reason	71.6	72.6	1.00	0.16	70.9	-0.70	0.51
Any technical quality reason	61.0	62.3	1.30	0.09	60.7	-0.30	0.83
Any non-technical quality reason	53.9	53.9	0.00	0.97	54.9	1.00	0.39
Any quality reason (technical or non-technical quality)	77.5	77.3	-0.20	0.78	77.9	0.40	0.66
**Households with no health insurance**							
Any access reason	70.9	71.8	0.90	0.11	68.4	-2.50	0.001
Any technical quality reason	60.5	62.5	2.00	0.0008	63.2	2.70	0.002
Any non-technical quality reason	55.2	56.4	1.20	0.03	58.7	3.50	<0.0001
Any quality reason (technical or non-technical quality)	77.7	78.5	0.80	0.09	80.8	3.10	<0.0001

URBAN HOUSEHOLDS							
	Households without hypertension	Households with hypertension only	Difference in %	chi-sq p-value	Households with hypertension and diabetes	Difference in %	chi-sq p-value
**All households**							
	%[a]	%[a]			%[a]		
Any access reason	65.1	65.9	0.80	0.09	63.2	-1.90	0.002
Any technical quality reason	57.0	58.9	1.90	0.0005	58.3	1.30	0.04
Any non-technical quality reason	59.3	60.6	1.30	0.01	61.7	2.40	0.0001
Any quality reason (technical or non-technical quality)	78.4	80.0	1.60	0.01	80.4	2.00	0.0001
**Poor households**							
Any access reason	68.2	68.5	0.30	0.83	68.3	0.10	0.97
Any technical quality reason	56.3	58.4	2.10	0.12	58.4	2.10	0.27
Any non-technical quality reason	55.8	57.0	1.20	0.38	58.7	2.90	0.12
Any quality reason (technical or non-technical quality)	76.6	78.2	1.60	0.16	80.2	3.60	0.02
**Households with no health insurance**							
Any access reason	65.8	67.3	1.50	0.008	63.6	-2.20	0.002
Any technical quality reason	56.8	58.5	1.70	0.004	57.7	0.90	0.17
Any non-technical quality reason	59.1	59.7	0.60	0.32	61.2	2.10	0.003
Any quality reason (technical or non-technical quality)	77.8	78.6	0.80	0.11	79.7	1.90	0.002
[a]Sampling weight proportion / standard deviation							
