# Supplementary material for: Reasons for low utilisation of public facilities among households with hypertension: analysis of a population-based survey in India
Source: BMJ Glob Health. 2018 Dec 20;3(6):e001002. doi: 10.1136/bmjgh-2018-001002 (PMC6307571; doi:10.1136/bmjgh-2018-001002)
Supplement: Supplementary data [file bmjgh-2018-001002supp003.pdf]

Supplemental Table 2. Demographic characteristics of households without valid biomarker data (N=41,890)

**Household demographics**	N	%[a]
Mean age of household members (mean, SD)	35.7	14.4
Mean age of head of household (mean, SD)	49.9	14.0
Any household member <5 years of age	9,220	22.4
Any household member ≥ 65 years of age	10,444	25.6
Mean household size (mean, SD)	4.0	2.0
Mean number of males in household (mean, SD)	2.0	1.2
Mean number of females in household (mean, SD)	2.0	1.2
Sex of head of household is male	34,868	83.6
Hindu religion (vs. all other religions)	31,293	79.5
Household has health insurance	9,985	28.6
Household has a below the poverty line card	15,150	41.2
Household has electricity	40,126	95.9
Household has a bicycle	14,392	38.9
Poor (lowest 40%)	15,274	38.2
Rural household location	21,690	53.1

[a]Sampling weight proportion / standard deviation
