# Supplementary material for: Reasons for low utilisation of public facilities among households with hypertension: analysis of a population-based survey in India
Source: BMJ Glob Health. 2018 Dec 20;3(6):e001002. doi: 10.1136/bmjgh-2018-001002 (PMC6307571; doi:10.1136/bmjgh-2018-001002)
Supplement: Supplementary data [file bmjgh-2018-001002supp004.pdf]

Supplemental Table 3. Private facility breakdown of facility utilization patterns of households with biomarker data in the DLHS-4 by chronic disease status, 2012-2013

	All households (N=336,305)		Households without hypertension (N=156,323)		Households with hypertension only (N=126,597)		Households with hypertension and diabetes (N=53,385)	
	N	%[a]	N	%[a]	N	%[a]	N	%[a]
Public primary health care facility	67,021	20.0	34,104	21.8	29,904	19.7	8,013	15.0
Public hospital	100,379	29.9	47,378	30.3	37,194	29.4	15,807	29.6
Private facility	162,382	48.3	71,226	45.6	62,289	49.2	28,867	54.1
Dispensary/clinic	59,495	17.7	27,666	17.7	22,669	17.9	9,160	17.2
Hospital	99,941	29.7	42,161	27.0	38,529	30.4	19,250	36.1
Ayush hospital/clinic	1,071	0.3	511	0.3	386	0.3	174	0.3
NGO or trust hospital/clinic	505	0.2	260	0.2	179	0.1	66	0.1
At home	780	0.2	369	0.2	300	0.2	111	0.2
Chemist/pharmacy	591	0.2	260	0.2	225	0.2	106	0.2
Other (non-medical shop, home treatment, other)	6,233	1.9	3,489	2.2	2,111	1.7	633	1.2
Missing	289	0.1	126	0.1	98	0.1	65	0.1
